# 

**Published:** 1865-05

**Authors:** 


### CHICAGO MEDICAL JOURNAL.

**Terms, $2.00 per Annum.**

### CONTENTS OF THE MAY NUMBER.

PAGE.

ORIGINAL COMMUNICATIONS.

Valedictory Address to the Graduating Class of the 22d Annual Session of Rush Medical College, Jan. 25th, 1865. By Prof. E. S. Carr, M. D. .......... 193

Clinical Lectures on Diseases of the Eye. By E. L. Holmes, M. D., of Chicago, Lecturer on Diseases of the Eye and Ear, in Rush Med. Col., etc.—Glaucoma .......... 206

Homœopathic Globules .......... 210

SELECTED.

On the Uses of Sugar and Lactic Acid in the Animal Economy. By Samuel Jackson, M. D., Emeritus Professor of the Institutes of Medicine in the University of Pennsylvania .......... 211

Notice of McPherson Hospital. By Col. T. P. Robb, State Sanitary Commissioner .......... 226

Last Hours of Abraham Lincoln. By C. S. Taft, Acting Ass't Surgeon U. S. A. .......... 227

EDITORIAL AND MISCELLANEOUS—The New Mission of the American Medical Association, &c. .......... 232

BOOK NOTICES AND REVIEWS .......... 238

